# Supplementary material for: Effect of the proximal secondary sphere on the self-assembly of tetrahedral zinc-oxo clusters
Source: Commun Chem. 2021 Sep 20;4:133. doi: 10.1038/s42004-021-00574-3 (PMC9814604; doi:10.1038/s42004-021-00574-3)
Supplement: Supplementary file 2 — Supplementary Information [file 42004_2021_574_MOESM2_ESM.pdf]

Supporting Information

Effect of the proximal secondary sphere on the self-assembly of tetrahedral zinc-oxo clusters

Michał Terlecki[1], Iwona Justyniak[2], Michał Leszczyński[1,2], Janusz Lewiński[1,2]*

[1]Faculty of Chemistry, Warsaw University of Technology, Noakowskiego 3, 00-664 Warsaw (Poland)
[2]Institute of Physical Chemistry, Polish Academy of Sciences, Kasprzaka 44/52, 01-224 Warsaw (Poland)

Table of contexts

1. Crystal data.. S3

2. Molecular and supramolecular structure analysis S5

3. NMR analysis S6

4. FTIR analysis S8

5. Phase transition analysis S9

6. TGA-DSC experiments S10

7. Nitrogen adsorption experiment.. S12

1. Crystal data

Table S1. Crystal data and structure refinement for comp **1**^{LT}.

Moiety formula	$C_{42}H_{42}N_{12}OZn_4$·5THF	
Empirical formula	$C_{62}H_{82}N_{12}O_6Zn_4$	
Formula weight	1352.87	
Temperature	100(2) K	
Wavelength	0.71073 Å	
Crystal system	Trigonal	
Space group	P 3 1 c	
Unit cell dimensions	a = 18.1390(7) Å	a= 90°.
	b = 18.1390(7) Å	b= 90°.
	c = 11.2920(3) Å	g = 120°.
Volume	3217.6(3) Å^3	
Z	2	
Density (calculated)	1.396 Mg/m^3	
Absorption coefficient	1.531 mm^{-1}	
F(000)	1412	
Crystal size	0.20 x 0.11 x 0.06 mm^3	
Theta range for data collection	3.159 to 27.472°.	
Index ranges	-23<=h<=23, -19<=k<=19, -13<=l<=14	
Reflections collected	8597	
Independent reflections	4556 [R(int) = 0.0480]	
Completeness to theta = 25.242°	99.5 %	
Absorption correction	Semi-empirical from equivalents	
Max. and min. transmission	0.912 and 0.817	
Refinement method	Full-matrix least-squares on F^2	
Data / restraints / parameters	4556 / 139 / 316	
Goodness-of-fit on F^2	1.072	
Final R indices [I>2sigma(I)]	R1 = 0.0397, wR2 = 0.0728	
R indices (all data)	R1 = 0.0475, wR2 = 0.0757	
Absolute structure parameter	0.04(2)	
Largest diff. peak and hole	0.49 and -0.36 e.Å^{-3}	

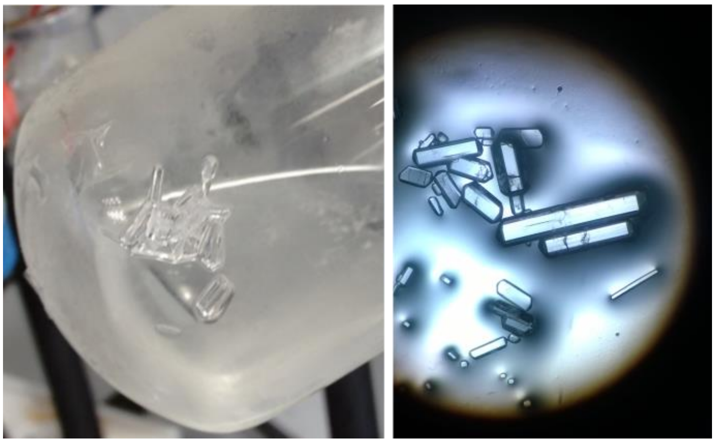

Fig. S1. Images of the single crystals of **1**^{LT}.

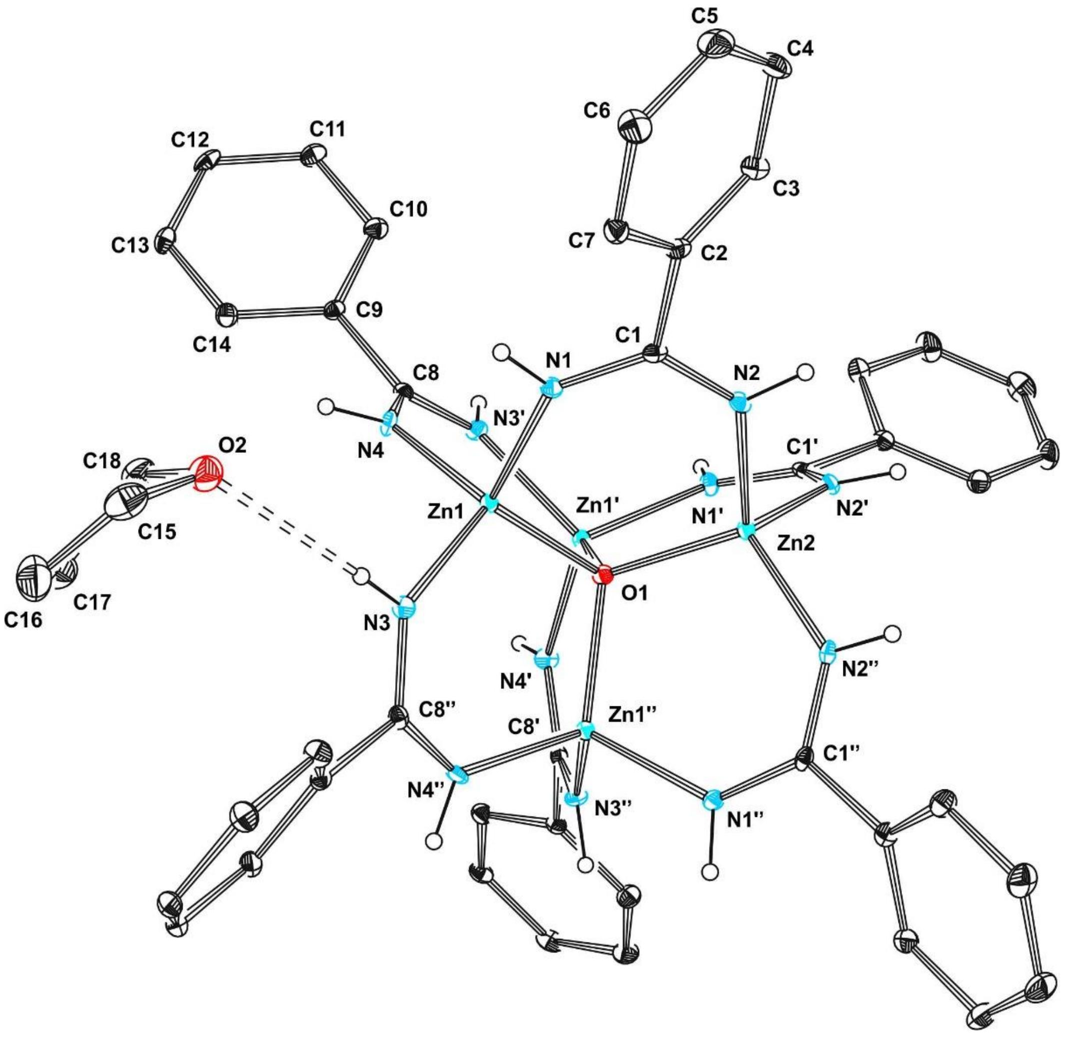

Fig. S2. Molecular structure of **1**^{LT} with thermal ellipsoids set at 40% probability. Hydrogen atoms have been omitted for clarity. Symmetry transformations used to generate equivalent atoms: (*-y*+1, *x-y*+1, *z*), (*-x*+*y*, *-x*+1, *z*), (*-x*+*y*, *-x*, *z*), (*-y*, *x-y*, *z*).

Table S2. Selected bond lengths [Å] and angles [°] for **1^{LT}**.

Zn1-O1	1.966(2)	Zn1-O1-Zn2	110.63(16)
Zn1-N1	2.004(4)	Zn1-O1-Zn1’	108.29(16)
Zn1-N3	1.986(4)	N1-Zn1-O1	111.3(2)
Zn1-N4	1.997(4)	N1-Zn1-N3	105.86(18)
Zn2-O1	1.957(6)	N1-Zn1-N4	104.51(19)
Zn2-N2	2.007(4)	N3-Zn1-O1	112.35(15)
C1-N1	1.321(6)	N4-Zn1-O1	112.60(16)
C1-N2	1.324(7)	N2-Zn2-O1	111.10(12)
C8’’-N3	1.315(7)	N2-Zn2-N2’	107.80(13)
C8-N4	1.317(7)		

Hydrogen bond	H···A	D···A	D−H···A	symmetry
N3−HN3···O2	2.33	3.170(6)	158.5	*x*, *y*, *z*

2. Molecular and supramolecular structure analysis

The molecular structure of **1^{LT}** comprises the tetrahedral $\{Zn_4O\}^{6+}$ core stabilized by six monoanionic benzamidinate μ_2-bridgeing ligands (Fig. S3a). The Zn centers in **1** adopt a distorted tetrahedral coordination environment with typical Zn-O and Zn-N bond lengths falling within a range of 1.957−1.966 Å and 1.986−2.004 Å, respectively. The amidinate functional groups in the ligands are symmetrical with C-N bond lengths of 1.321−1.324 Å. Interestingly, while in the benzoic and benzamidate analogs, $[Zn_4O(L^{OO})_6]$ and $[Zn_4O(L^{NO})_6]$, the phenyl rings in the ligands are essentially coplanar with the functional carbonyl groups, in the molecular structure of **1** they are twisted from the planes of amidinate functionalities by ca. 40-45° (the corresponding C_{am}-N_{am}-C_{Ar}-C_{Ar} torsion angles are in the range of 39.3-47.2°, Fig. S3c). In the crystal structure of **1^{LT}**, the zinc-oxo clusters form **1**·5THF solvates with one H-bonded THF molecule on each vertex of the tetragonal core (O···H distances are 2.238 and 2.333 Å) (Fig. S3b) and one additional THF molecule included in 1D open channel in the supramolecular framework (Fig. 3h).

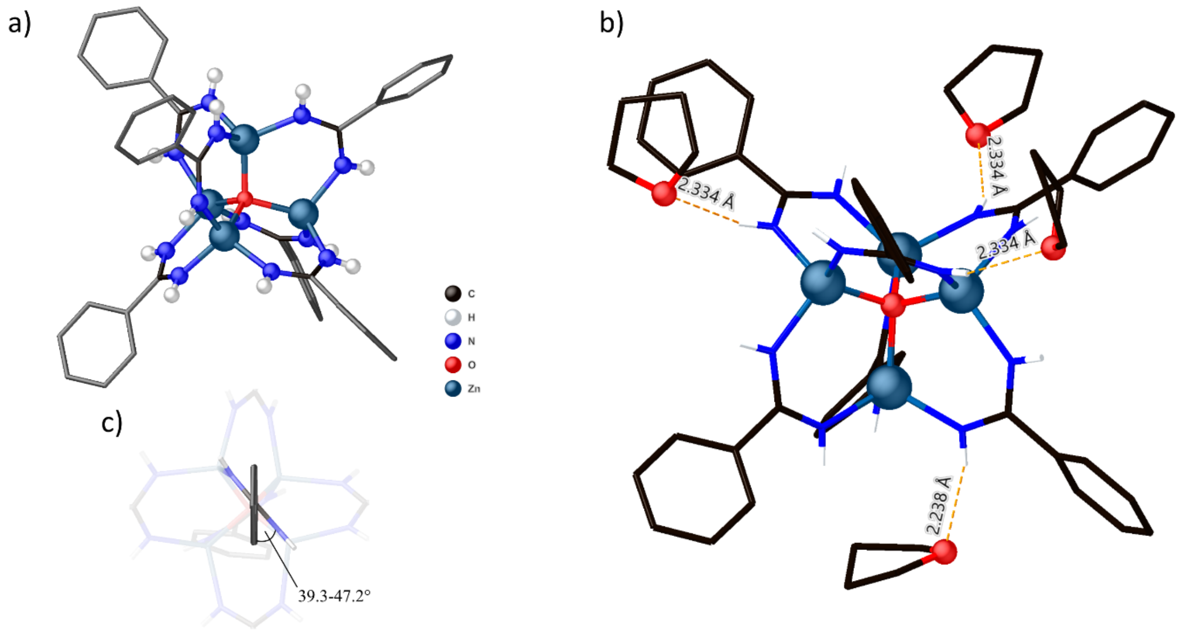

Fig. S3. Molecular structure of $\mathbf{1}^{LT}$ (a), and view on the noncovalent **1**·4THF solvate (b) and conformation of the benzamidinate ligands (c).

3. NMR analysis

^{1}H and ^{13}C NMR spectra of $\mathbf{1}^{LT}$ and $\mathbf{1}^{RT}$ in d_8-THF are essentially identical. The ^{1}H NMR spectrum shows two multiples at 7.59 and 7.28 ppm associated with the monosubstituted phenyl ring and a narrow singlet at 4.71 ppm from N-bonded hydrogen atoms. The ^{13}C NMR spectrum shows a set of signals from tertiary carbon atoms of the phenyl ring in the range of 125-130 ppm and two signals with the higher chemical shifts at 144.79 and 174.64 ppm related to the quaternary aromatic and amidinate carbon atoms, respectively.

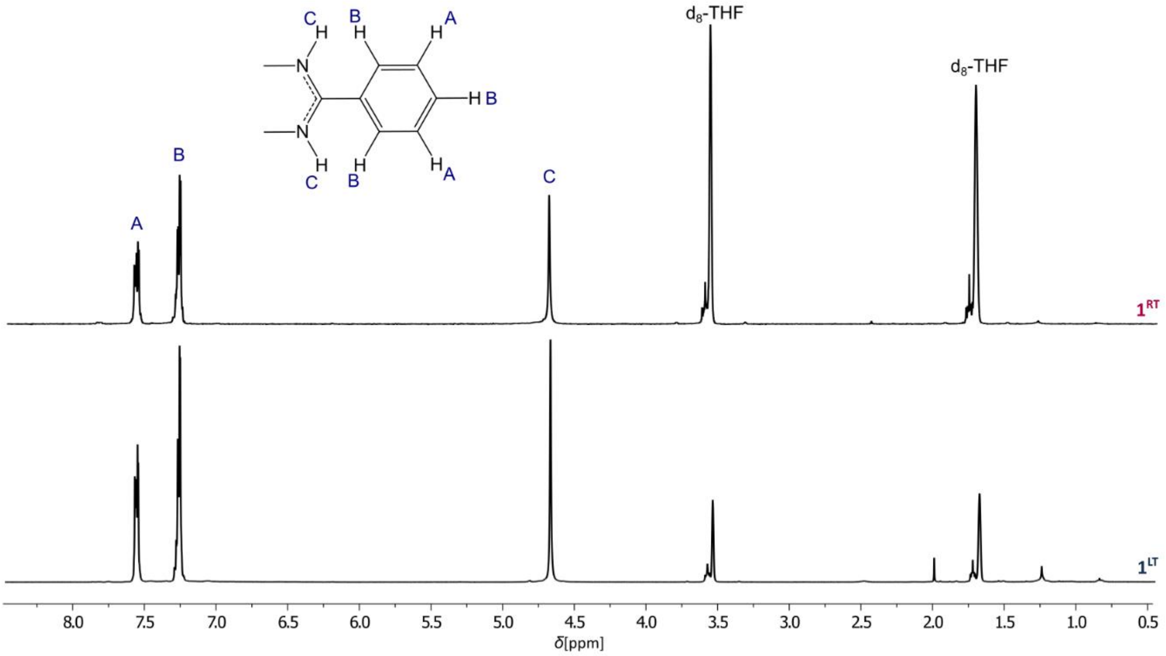

Fig. S4. ^{1}H NMR in d_8-THF of $\mathbf{1}^{RT}$ and $\mathbf{1}^{LT}$.

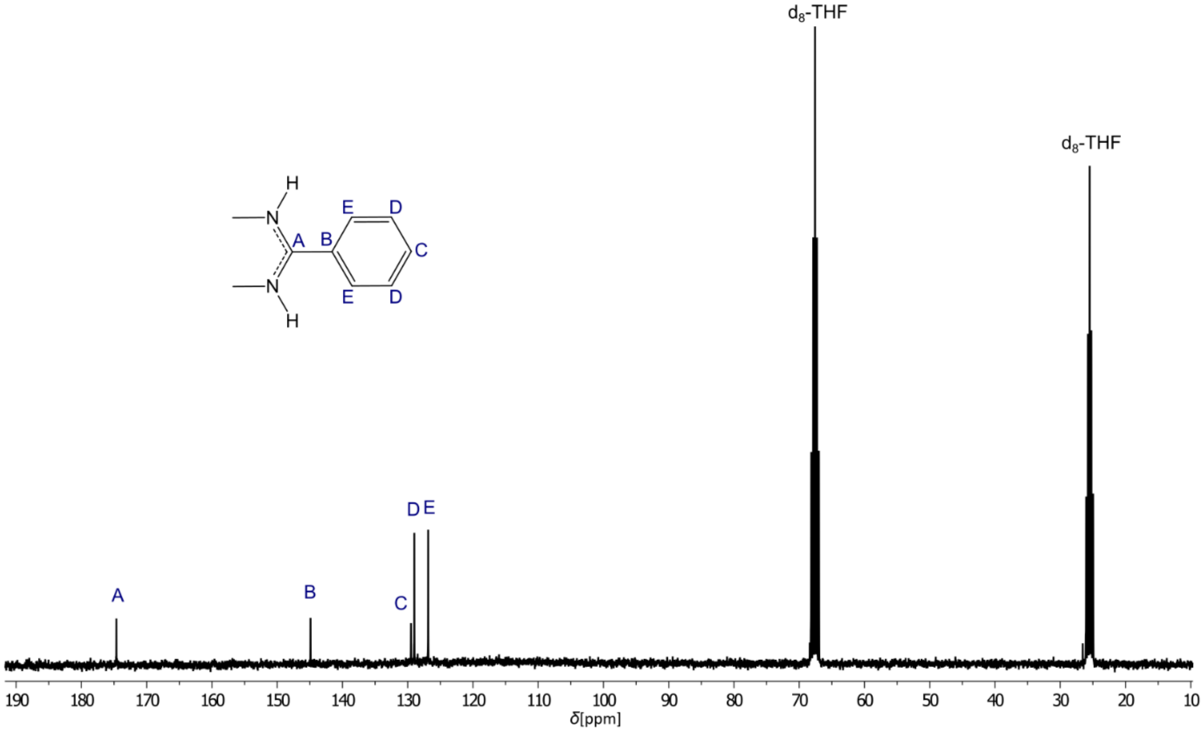

Fig. S5. ^{13}C NMR in d_8-THF of **1**$^{\mathbf{RT}}$.

4. FTIR analysis

The solid-state FTIR spectra of both $\mathbf{1}^{LT}$ and $\mathbf{1}^{RT}$ phases show bands at 3366 and 1593 cm^{-1} associated with $\nu_{(N\text{-}H)}$ and $\nu_{(C\text{-}N)}$ stretching vibrations of amidine groups, and bands at 3058, 1560, 1512, 1477, 698 cm^{-1}, which are characteristic for the monosubstituted aromatic ring. Interestingly, the $\nu_{(N\text{-}H)}$ band in the spectrum of $\mathbf{1}^{LT}$ is clearly split, which indicates the involvement of N-H groups in the formation of hydrogen bonds. Additional bands at 2970, 2857, 1061, and 905 cm^{-1} indicate the presence of THF molecules in the crystal lattice of both phases. The THF signals are significantly more intensive in the case of $\mathbf{1}^{LT}$, which is consistent with the estimated stoichiometry of both solvomorphic forms (**1**·5THF and **1**·0.86THF for $\mathbf{1}^{LT}$ and $\mathbf{1}^{RT}$, respectively).

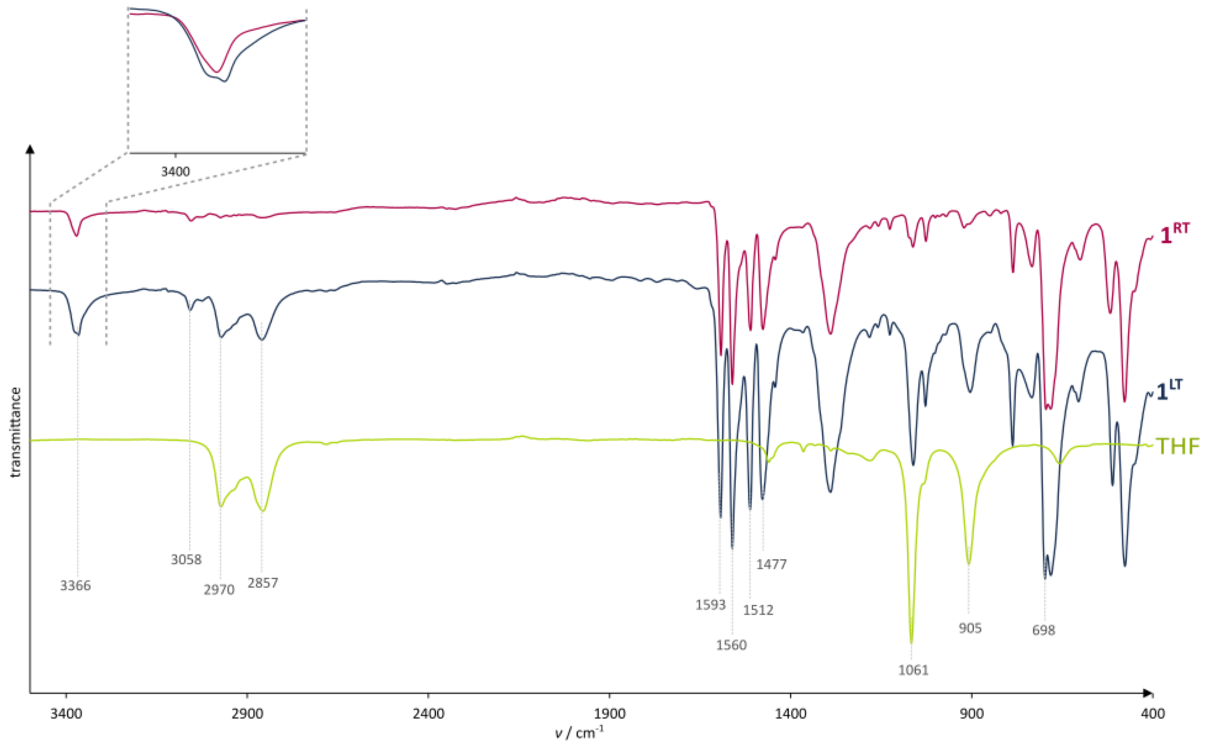

Fig. S6. FTIR spectra of $\mathbf{1}^{RT}$, $\mathbf{1}^{LT}$, and THF.

5. Phase transition analysis

Monocrystals of **1**^{LT} isolated at -20°C were stable up to about 10-20°C, where the irreversible phase transition into the phase **1**^{RT} occurs, which is accompanied by the fragmentation of the starting single crystal material (Fig. S8). Crystals of **1**^{LT} were stable during long drying under vacuum at -10°C and the resulting dry material exhibited the same phase transition to **1**^{RT} under about 10°C (Fig. S9). Furthermore, the same transformation is observed when the crystals of **1**^{LT} are covered by THF (Fig. S9). This indicates that the phase transition is rather induced by thermal dissociation of the weak N-H···THF hydrogen bonds then simple solvent evaporation.

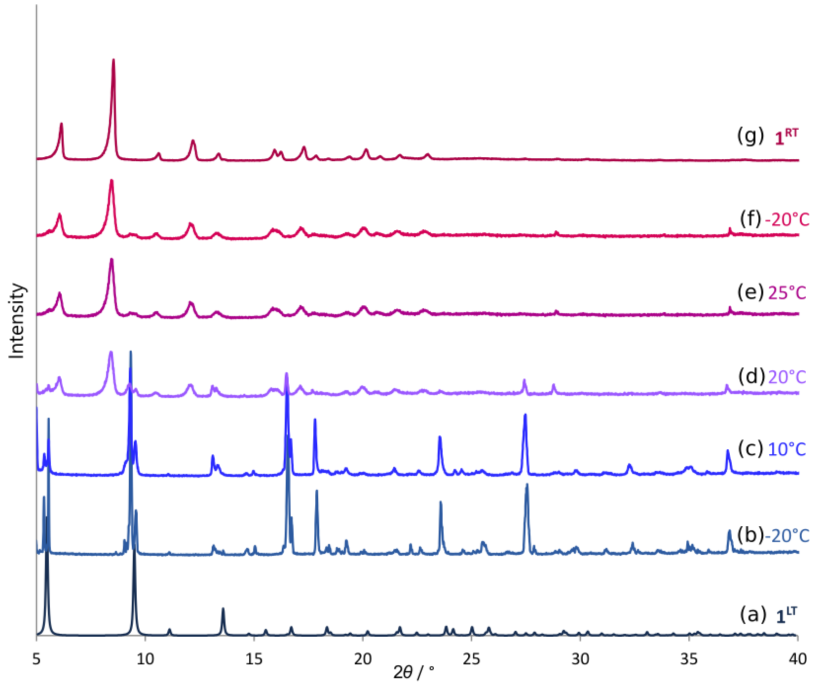

Fig. S7. PXRD patterns generated from the crystal structure of **1**^{LT} (a), measured for crystals of **1**^{LT} heated from -20°C to 25°C (b-e) and then cooled to -20°C (f), and measured for phase **1**^{RT} obtained in the room temperature synthesis (g). [Note, that high peak intensities for reflections (101), (102), (302) and (402) in experimental powder patterns of **1**^{LT} are an effect of crystal orientation]

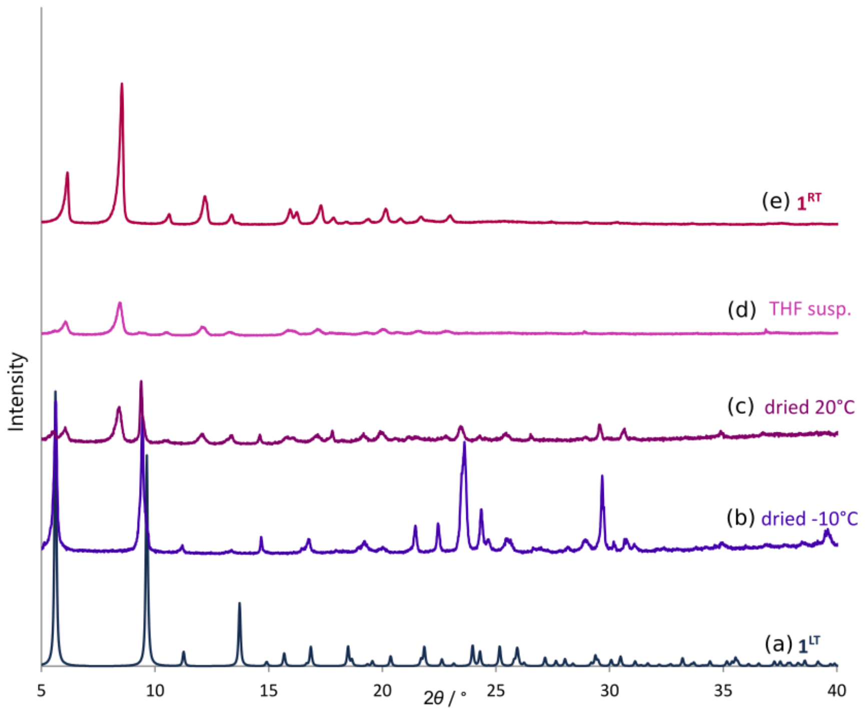

Fig. S8. PXRD patterns: generated from the crystal structure of **1**^{LT} (a), measured for crystals of **1**^{LT} dried under vacuum for 5h at -10°C (b) and then heated to 20°C (c), measured for crystals of **1**^{LT} covered by THF at 20°C (d), and measured for phase **1**^{RT} obtained in the room temperature synthesis (e).

6. TGA-DSC experiments

DSC curve of **1**^{LT} shows an endothermic peak above about 30°C associated with a phase transition into **1**^{RT} form. Above this temperature, the TGA-DSC plots of both **1**^{LT} and **1**^{RT} are essentially identical showing two mass decreases, one between 70-120°C associated with the solvent evaporation and one above 200°C associated with the decomposition of the zinc-oxo complex. The sample of **1**^{LT} was dried under vacuum at -10°C, which seems insufficient to remove all the THF molecules (the remaining solvent content is still about 7.40%). The solvent evaporation in **1**^{RT} occurs significantly above the boiling point of THF, which indicates tight encapsulation of the guest into the host supramolecular network. Moreover, the estimated THF content in the sample is in good agreement with the stoichiometry **1**·0.86THF estimated based on the elemental analysis (calc. 5.88%, exp. 5.84%).

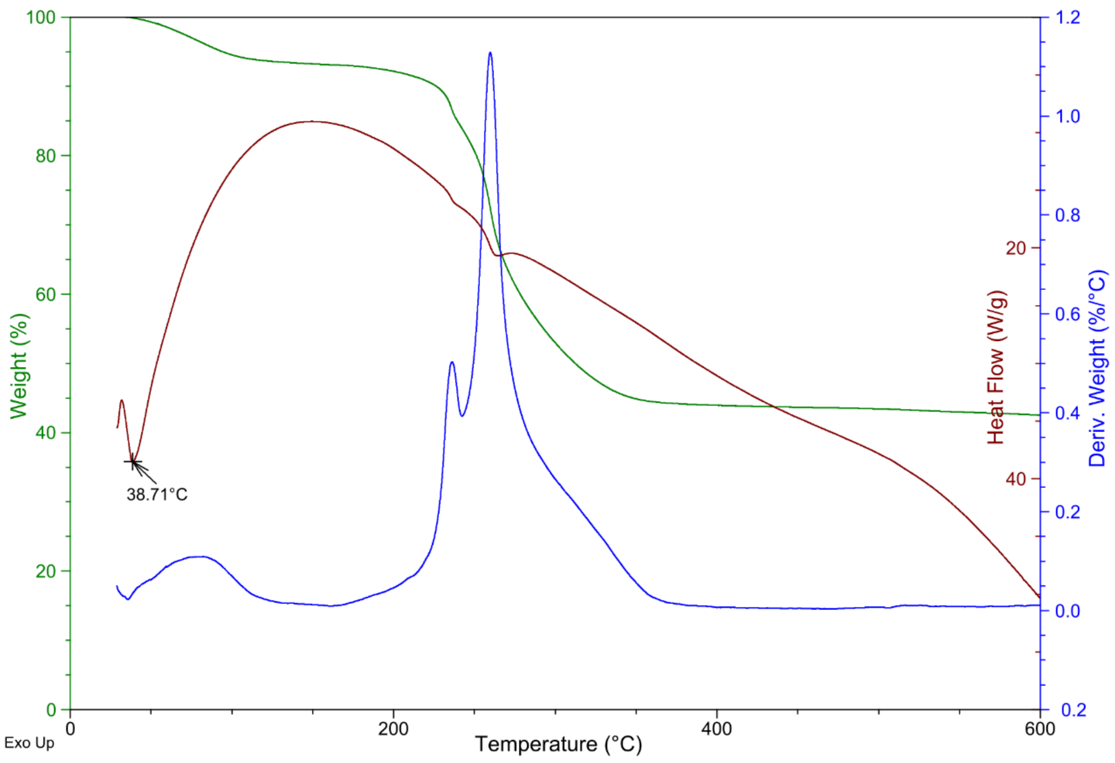

Fig. S9. TGA-DSC plot for **1**^{LT}.

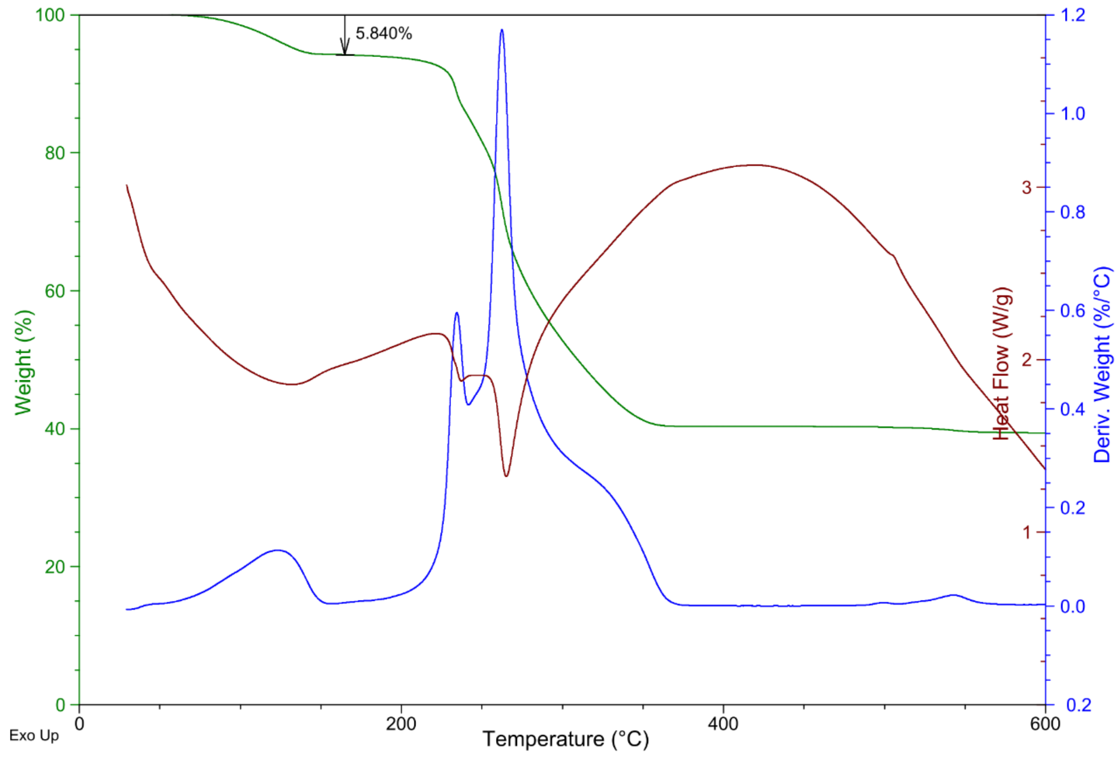

Fig. S10. TGA-DSC plot for **1**^{RT}.

7. Nitrogen adsorption experiment

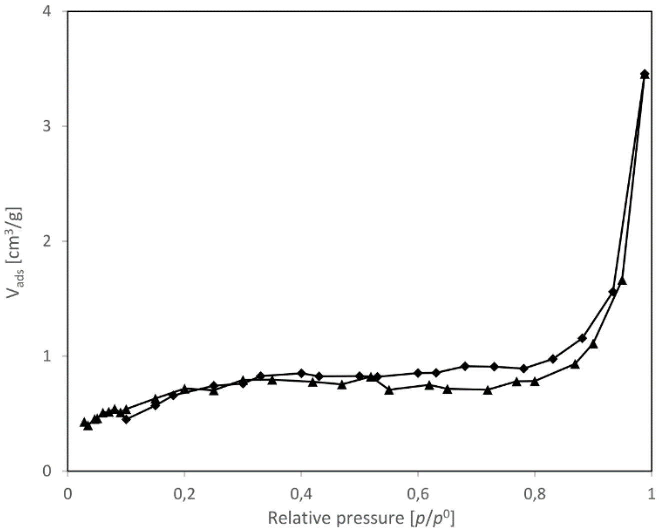

Fig. S11. Nitrogen adsorption–desorption isotherm at 77 K for $\mathbf{1^{LT}}$.
