# Supplementary material for: Effect of the proximal secondary sphere on the self-assembly of tetrahedral zinc-oxo clusters
Source: Commun Chem. 2021 Sep 20;4:133. doi: 10.1038/s42004-021-00574-3 (PMC9814604; doi:10.1038/s42004-021-00574-3)
Supplement: Supplementary file 3 — Description of Additional Supplementary Files [file 42004_2021_574_MOESM3_ESM.pdf]

Description of Additional Supplementary Files

File Name: Supplementary Data 1

Description: X-ray crystallography data of $\mathbf{1}^{LT}$
